# Impaired Hypothalamic Microglial Activation in Offspring of Antibiotic-Treated Pregnant/Lactating Rats Is Attenuated by Prebiotic Oligofructose Co-Administration

**DOI:** 10.3390/microorganisms8071085

**Published:** 2020-07-21

**Authors:** Nicole A. Cho, Alissa C. Nicolucci, Teja Klancic, Weilan Wang, Keith A. Sharkey, Richelle Mychasiuk, Raylene A. Reimer

**Affiliations:** 1 Faculty of Kinesiology, University of Calgary, Calgary, AB T2N 1N4, Canada; nacho@ucalgary.ca (N.A.C.); alissa.c.nicolucci@gmail.com (A.C.N.); klancic.teja@gmail.com (T.K.); weilan.wang@ucalgary.ca (W.W.); 2 Hotchkiss Brain Institute and Snyder Institute of Chronic Diseases, Department of Physiology and Pharmacology, University of Calgary, Calgary, AB T2N 1N4, Canada; ksharkey@ucalgary.ca; 3 Department of Neuroscience, Central Clinical School, Monash University, Melbourne 3004, Australia; richelle.mychasiuk@monash.edu; 4 Department of Biochemistry & Molecular Biology, University of Calgary, Calgary, AB T2N 1N4, Canada

**Keywords:** antibiotics, prebiotics, oligofructose, gut microbiota, behavior

## Abstract

Microbial colonization of the gut early in life is crucial for the development of the immune and nervous systems, as well as influencing metabolism and weight gain. While early life exposure to antibiotics can cause microbial dysbiosis, prebiotics are non-digestible substrates that selectively promote the growth of beneficial gut microbiota. Our objective was to examine the effects of dietary prebiotic administration on the consequences of maternal antibiotic intake on offspring body weight, behavior, and neuroimmune responses later in life. Sprague-Dawley rat dams were given low-dose penicillin (LDP), prebiotic fiber (10% oligofructose), or both, during the third week of pregnancy and throughout lactation. Anxiety-like behavior, weight gain, body composition, cecal microbiota composition, and microglial responses to lipopolysaccharide (LPS) were assessed in offspring. Male and female prebiotic offspring had lower body weight compared to antibiotic offspring. Maternal antibiotic exposure resulted in lasting effects on select offspring microbiota including a lower relative abundance of *Streptococcus, Lactococcus*, and *Eubacterium* at 10 weeks of age. Maternal antibiotic use impaired microglial response to LPS in the hypothalamus compared to control, and this phenotype was reversed with prebiotic. Prebiotic fiber warrants further investigation as an adjunct to antibiotic use during pregnancy.

## 1. Introduction

The establishment and development of the infant gut microbiota is a dynamic and complex process affected by perinatal conditions including maternal diet, mode of delivery, and antibiotic exposure [1,2]. Although there is some debate whether the placenta houses a microbiome, it is accepted that the first major exposure of infants to microbiota is during labor and birth [2]. Transfer of microbiota to an infant via vertical transmission from the mother influences the development of the immune system, which in turn impacts brain development and function [3,4,5]. There is a critical developmental window occurring over the first weeks of life in rodents and few years of life in humans whereby disruptions in the gut microbial community (termed microbial dysbiosis) have long-lasting impacts on brain development [6]. While the exact mechanisms by which microbial dysbiosis alters brain function remain to be elucidated, the pathways mediating these effects are part of the microbiota-gut-brain axis [7]. Microglia, the resident immune cells of the brain, are critical in early life for proper brain development through synaptic pruning, remodeling, supporting neurogenesis, and neuron survival [8]. Microglia-mediated synaptic pruning has been shown to be altered following antibiotic exposure in mice, leading to deficits in fear extinction learning, which could only be reversed by selective re-establishment of the microbiota before weaning [9]. Germ-free (GF) mice show defects and immaturity in their microglia, a defective phenotype that was reversed through supplementation with short-chain fatty acids (SCFA), a by-product of microbial metabolism of dietary fibers [8]. Similarly, supplementation with the prebiotic fiber, oligofructose-enriched inulin, was shown to improve age-related impairment of microglia, as well as reduce anxiety-like behavior and improve learning in young adult mice [10].

Early life and old age are periods when the microbiota have increased susceptibility to perturbation, have reduced diversity, and lack resiliency when faced with adverse environmental factors such as antibiotics or an unhealthy diet [7,11]. In early life (intrauterine period, during delivery, and until three years of age), exposure to antibiotics can be particularly harmful over the long term [4,12]. While antibiotics are life-saving medications that can prevent and treat serious illness, they can also disrupt the beneficial commensal microbial community that is essential to an infants’ development. Antibiotics are reported to comprise up to 80% of prescriptions in pregnancy, and penicillins are the most widely prescribed class of antibiotics [13]. Intrapartum antibiotics, commonly used to prevent infection associated with caesarean-section birth and/or to block the vertical transmission of group B *Streptococcus* (GBS) during labor and delivery, are associated with reduced bacterial diversity, and lower abundance of beneficial *Bifidobacterium* and *Lactobacillus* in the infant gut [14]. Prenatal antibiotic use is also associated with increased risk of childhood asthma [15] and obesity [16]. Although other conditions have been associated with early life antibiotic exposure, some of the data is conflicting such as with autism spectrum disorder (ASD) [17], while other emerging evidence suggests that neonatal antibiotic use increases the risk of developing functional gastrointestinal disorders including infantile colic and regurgitation [18]. Given that antibiotic use during pregnancy and/or intrapartum will continue to be clinically indicated, it is crucial to identify ways in which the risks of this early life antibiotic exposure, particularly on the developing brain and behavior, can be mitigated.

A potential safe dietary intervention to ameliorate the gut microbiota-associated effects of antibiotics could be dietary supplementation with prebiotics. Prebiotics are non-digestible substrates that are metabolized by host microorganisms, conferring a health benefit [19]. Prebiotics, particularly inulin and oligofructose, have been shown to increase *Bifidobacterium*, which has beneficial metabolic effects, such as reducing body fat, serum triglycerides, and the proinflammatory marker interleukin-6 (IL-6) in children with overweight or obesity [20]. A previous study also found that prebiotic supplementation was able to reduce stress-induced corticosterone release and reduce depression-like and anxiety-like behaviors in mice [21]. There are limited studies in clinical populations looking at prebiotic use during pregnancy, but a systematic review found there was no positive or negative effect of prebiotic use on preterm birth or adverse birth outcomes [22]. We have previously found that prebiotic use in rats during pregnancy was associated with improved fertility and weight loss [23]; however, to our knowledge there are no studies that have examined whether prebiotics can improve microbiota-gut-brain axis function in conjunction with maternal antibiotic use. Our objective was to examine the effect of prebiotic co-administration with maternal antibiotic use on offspring body composition, behavior, and neuroimmune responses. We hypothesized that supplementing maternal antibiotic intake with prebiotic diet would attenuate offspring excess weight gain associated with consumption of a high fat/sucrose diet, reduce anxiety-like behavior, improve learning, and alter microglial activation in response to a peripheral immune challenge. Here we show that maternal antibiotic administration impaired microglial reactivity and increased proinflammatory activity in the brain of their offspring. Co-administration of prebiotic oligofructose mitigated some but not all of these detrimental effects; most notably, prebiotic supplementation reversed the immature microglial phenotype.

## 2. Materials and Methods 

### 2.1. Animals and Experimental Design

Seventy Sprague-Dawley rats (10 weeks old, *n* = 60 females, *n* = 10 males) were obtained from Charles River Laboratories (Saint Constant, QC, Canada) and were housed on a 12 h light-dark cycle in a temperature (20–22 °C) and humidity (41–60%) controlled room. After a two-week acclimatization period, rats were mated and randomized into one of four groups: (1) Control [CT], (2) Antibiotic [AB] (low dose penicillin G (LDP); 1 µg/g/day; Sigma Aldrich, Oakville, ON, Canada), (3) Prebiotic [PR] (10% oligofructose (OFS) diet; 10% wt/wt, Orafti P95, BENEO-Orafti Inc.), or (4) or Antibiotic + Prebiotic (AB + PR) (LDP + 10%OFS diet). During the third week of pregnancy and throughout lactation, dams consumed LDP via their drinking water as described previously [24,25]. Oligofructose was provided at a dose of 10% which has been widely used in the literature and has been shown to reduce fat mass and inflammation and selectively promote the growth of *Bifidobacterium* [26,27,28]. LDP added to drinking water was calculated based on average water intake. The dose of penicillin (1 µg/g/day) was based on the range approved for use in agriculture by the US Food and Drug Administration (FDA) [24,25]. To achieve the 1 µg/g dose, water intake was measured and the concentration of penicillin adjusted twice weekly according to dams’ body weight. The CT and AB dams consumed control AIN-93G diet (Dyets Inc., Bethlehem, PA, USA), while the PR and AB + PR dams consumed the AIN-93G diet supplemented with 10%OFS during the third week of pregnancy and throughout lactation. 

To avoid differences in nutritional exposure due to varied litter size, all litters were culled to 10 pups (*n* = 5 males, *n* = 5 females, where possible) within 24 h of parturition. Pups from the same dam were counted as *n* = 1. Forty-seven dams successfully gave birth to full litters of 10 offspring, resulting in a total of 10 CT, 11 AB, 13 PR, and 13 AB + PR successful litters. Maternal and pup body weight was measured weekly. All pups were weaned at 3 weeks of age onto a control diet (AIN-93G) until 5 weeks of age followed by a high-fat/high-sucrose diet (HFS) (diet #102412; Dyets, Bethlehem, PA, USA) until 10 weeks of age, which served to create an immune challenge to unmask an activated microglial phenotype [29,30]. Ethical and study approval was given by the University of Calgary Animal Care Committee (Protocol #AC15-0079) and conformed to the *Guide to the Care and Use of Laboratory Animals* by the Canadian Council on Animal Care.

### 2.2. Food Intake

Offspring food intake was recorded when pups were 4 and 6 weeks of age, for five consecutive days at each time point. Food intake was measured daily at the respective timepoints by weighing feed cups and is reported as average kcal/day. From 3–4 weeks of age, offspring consumed AIN-93G diet, which has an energy density of 3.76 kcal/g. From 5–10 weeks of age, offspring consumed the HFS diet, which has an energy density of 4.60 kcal/g.

### 2.3. Behavioral Testing

At 8 weeks of age, all groups underwent the Elevated Plus Maze (EPM) test and at 9 weeks, they underwent the Novel Context Mismatch (NCM) test as previously described [31,32]. All behavioral tests occurred during the light phase between 12:00 pm and 7:00 pm. The EPM consists of an elevated plexiglass maze with two open arms and two closed arms, intersecting to form a plus shape. The rodent’s behavior in this task reflects the preference for the animal to remain protected in closed arms and an innate curiosity and motivation to explore novel environments in the open arms [33]. For NCM, rats are exposed to two distinct contexts or enclosures with two identical objects for 5 min each day, for three consecutive days. On the probe day, they could explore each familiar context, followed by a modified context, where one of the identical objects from Context A was placed in Context B and vice versa. The NCM tests for spatiotemporal memory and learning [34]. For both tests, all objects, context bins, and equipment were cleaned with Virkon between each session. A research associate blinded to experimental conditions scored the EPM and NCM videos.

### 2.4. Body Weight and Composition

Offspring body weight was measured weekly throughout the study. At 10 weeks of age, offspring were lightly anaesthetized with isoflurane and body composition was assessed via a dual energy x-ray absorptiometry (DXA) scan with software for small animals (Hologic ODR 4500; Hologic Inc., Bedford, MA, USA).

### 2.5. LPS Challenge

At 10 weeks of age, offspring underwent an acute peripheral inflammatory challenge. Lipopolysaccharide (LPS) (100 µg/kg body weight [35]; Sigma-Aldrich, Oakville, ON, Canada) was diluted with sterile phosphate-buffered saline (PBS) and injected intraperitoneally. This dose of LPS activates the immune system but does not cause septic shock [35]. Control animals received an injection of sterile PBS. Both groups were injected with 1 ml/kg body weight of fluid. All tissues were collected 24 h after injection.

### 2.6. Tissue Collection for Gene Expression

At 10 weeks of age, 24 h after an LPS or saline injection, in the morning from 8:00–10:00 a.m. after an overnight fast, animals were euthanized with an overdose of anesthetic (isoflurane) and the aorta cut. Samples (cecal matter and proximal colon tissue) were collected, weighed, and snap frozen in liquid nitrogen. Brain samples (hypothalamus and hippocampus) were collected and placed on dry ice. All tissues were stored in −80 °C until analysis. Total RNA was extracted from the proximal colon, hypothalamus, and hippocampus, cDNA was synthesized and RT-PCR performed as previously described [36]. ß-actin was confirmed to be a suitable reference gene that remained unchanged in response to treatment, and differences in mRNA levels were calculated using the 2^−ΔΔCT^ method. Genes investigated (CD11b, glial fibrillary acidic protein (GFAP), ionized calcium binding adaptor molecule 1 (Iba-1), interleukin 1 beta (IL-1β), dopamine receptor D1 (Drd1), dopamine receptor D2 (Drd2), toll-like receptor 4 (TLR4), tumor necrosis factor (TNF), and CCL2) were chosen for their role in neuroinflammation most notably as macrophage markers (CD11b, CCL2, TLR4) and others for their influence on microglial activity [37]. In the proximal colon, occludin and the tight junction protein zonula-occludens-1 (ZO-1) were chosen as they are involved in the maintenance of epithelial integrity and the regulation of gut permeability [38].

### 2.7. Immunohistochemistry

A small subgroup of animals was used for microglia analysis (*n* = 3–6/group). At 10 weeks of age 24 h after an LPS injection, rats were transcardially perfused with cold saline followed by 4% paraformaldehyde (PFA) in PBS (pH 7.3). Brains were removed and fixed in 4% PFA at 4 °C overnight, and cryoprotected with 20% sucrose and 30% sucrose in PBS at 4 °C for the following two nights. Specimens were embedded in OCT compound (VWR International, Mississauga, ON, Canada) and sectioned in the coronal plane on a cryostat (40 µm). Floating sections were collected with reference to the rat brain atlas [39] which contained the paraventricular nucleus (PVN) of the hypothalamus. Sections were immunolabeled for the presence of mononuclear phagocytes (microglia and macrophages) as previously described [40]. Sections were incubated with primary Iba-1 antibodies (48 h; 1:1000; Wako Pure Chemical Industries, Osaka, Japan), followed by the secondary antibody (2 h; 1: 400, donkey anti-rabbit IgG [CY3]; Jackson Immunoresearch Laboratories).

### 2.8. Image Acquisition and Quantification

Sections were mounted and observed using a Zeiss Axioplan fluorescence microscope using a 10× objective (Carl Zeiss, Jena, Germany) and grey-scale images were captured by digital camera (Qimaging, Surrey, BC, Canada). Cell counts and area measurements were performed using the software ImageJ (NIH, USA) per 1.77 mm^2^ of selected area [41]. For microglia complexity analysis, images were converted to 8-bit files, and manually outlined and isolated. This image was converted to binary and skeletonized [42]. Skeletonized microglia were analyzed using the Sholl Analysis plugin provided by Fiji/ImageJ by an individual blinded to the treatment groups, as previously described [43].

### 2.9. Cecal 16S rRNA Illumina Sequencing

Cecal contents were collected at 10 weeks of age. Using ~250 mg of cecal matter, total bacterial DNA was extracted with a bead beating step using a FastDNA Spin Kit for feces (MP Biomedicals) and quantified (PicoGreen kit, Invitrogen). DNA samples were diluted to 20 ng/mL for sequencing. The amplicons of the 16S rRNA gene V3–V4 region were sequenced with the MiSeq Illumina platform (2 × 300; Illumina Inc., San Diego, CA, USA) at the Centre for Health Genomics and Informatics (University of Calgary, Calgary, AB, Canada) [44]. Quality check and denoising of demultiplexed reads were done in QIIME2 (QIIME2-2020.2) [45] by using ‘dada2 denoise-paired workflow’ with parameters: --p-trim-left-f 17 --p-trim-left-r 21 --p-trunc-len-f 270 --pt-trunc-len-r 240 [46]. Sequence variants with low abundance were filtered out by ‘feature-table filter-features’ plugin with parameter --p-min-frequency 10. Taxonomy was assigned to representative sequences by using Silva132. Classifier α and β diversity were calculated after rarefying depth to 24,390 sequences per sample. All sequences were uploaded into the National Centre for Biotechnology Information (NCBI) Sequence Read Archive (SRA) and can be found under accession number PRJNA646513.

### 2.10. Statistical Analysis

All data are presented as means ± standard error of the mean (SEM). Outcomes with a single time point (e.g., microglia parameters, body fat, etc.) were analyzed using a two-way analysis of variance (ANOVA) to determine the main effects of maternal diet, sex, and their interaction. If there was a significant main effect of sex, a one-way ANOVA with Tukey’s post hoc was performed separately for males and females. Outcomes with repeated timepoints (e.g., body weight) were analyzed using repeated-measures ANOVA, with time as a within-subject factor and maternal diet and sex as between-subject factors. If there was a significant main effect of sex, male and female data were analyzed separately. If there was a significant maternal diet × time interaction, a one-way ANOVA with Tukey’s post hoc test was used to assess differences between treatment groups. α-diversity was analyzed by Kruskal-Wallis rank-sum test. Community structure of cecal microbiota was compared based on weighted UniFrac distance matrix using analysis of similarity (ANOSIM) with 999 permutations. Slight correlation between factors and community dissimilarities was considered when R value <0.3, whereas R >0.3 was considered a strong correlation. Differences in sequences relative abundance was determined by using Kruskal test with Wilcox test for pairwise comparison. *p*-values of 0.05 with Bonferroni-adjustment were considered significant.

## 3. Results

### 3.1. Offspring Anthropometrics

**Figure 1:**
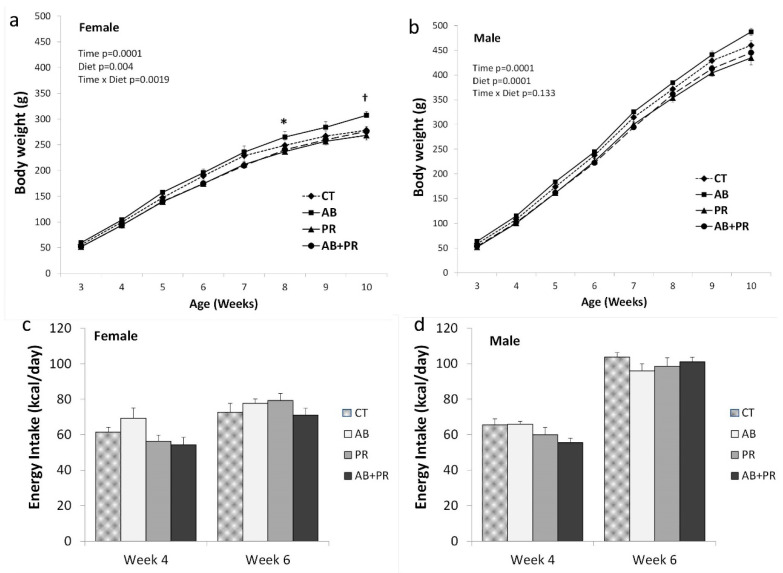
(**a**) Female offspring body weight; (**b**) male offspring body weight; (**c**) female offspring energy intake; (**d**) male offspring energy intake. Values are mean ± SEM (*n* = 10–13/group). * AB significantly different from PR (*p* = 0.01) and AB + PR (*p* = 0.027). † AB significantly different from PR (*p* = 0.004) and AB + PR (*p* = 0.029) with a trend compared to CT (*p* = 0.077). Significant diet effect for male body weight is AB heavier than PR (*p* = 0.0001) and AB + PR (*p* = 0.001). CT: control; AB: antibiotic; PR: prebiotic; AB + PR: antibiotic plus prebiotic.

There was a significant effect of sex (*p* < 0.0001) on offspring body weight. Therefore, females (Figure 1a) and males (Figure 1b) were analyzed separately. For females, there was a significant effect of time (*p* = 0.0001), diet (*p* = 0.004), and time × diet (*p* = 0.0019) for body weight. At 8 weeks of age, AB females were significantly heavier than PR (*p* = 0.01) and AB + PR (*p* = 0.027). At 10 weeks of age, AB females were significantly heavier than PR (*p* = 0.004) and AB + PR (*p* = 0.029) with a trend compared to CT (*p* = 0.077). For males, there was a significant effect of time (*p* = 0.0001) and diet (*p* = 0.0001). The significant diet effect was reflected in higher body weight in AB males compared to PR (*p* = 0.0001) and AB + PR (*p* = 0.001). There was a significant effect of sex (*p* = 0.0001) on energy intake; therefore, males and females were analyzed separately (Figure 1c,d). There were no significant differences between diets for energy intake at 4 and 6 weeks of age, although in females at 4 weeks of age there was a trend (*p* = 0.073) for AB to consume more energy than AB + PR.

There was a significant effect of sex for fat mass, lean mass, bone mineral content (BMC), bone mineral density, cecum weight, and brain weight (all *p* = 0.0001) and percent body fat (*p* = 0.025); therefore, females and males were analyzed separately (Table 1). In females at the end of the study, fat mass was higher in AB compared to PR (*p* = 0.014) and AB + PR (*p* = 0.012). Percent body fat was significantly higher in AB compared to AB + PR (*p* = 0.038). There was a trend (*p* = 0.056) for brain weight expressed per body weight to differ in females with AB having the lowest brain weight. In males, AB had significantly higher total body weight compared to PR (*p* = 0.008) and AB + PR (*p* = 0.048). Lean mass was significantly higher in AB compared to PR (*p* = 0.006) although when expressed as percent lean mass, PR had 78.9% and AB had 78.0% lean body mass. Cecum weight expressed per body weight was significantly different with PR being higher than CT (*p* = 0.042).

### 3.2. Behavioral Tests

**Figure 2:**
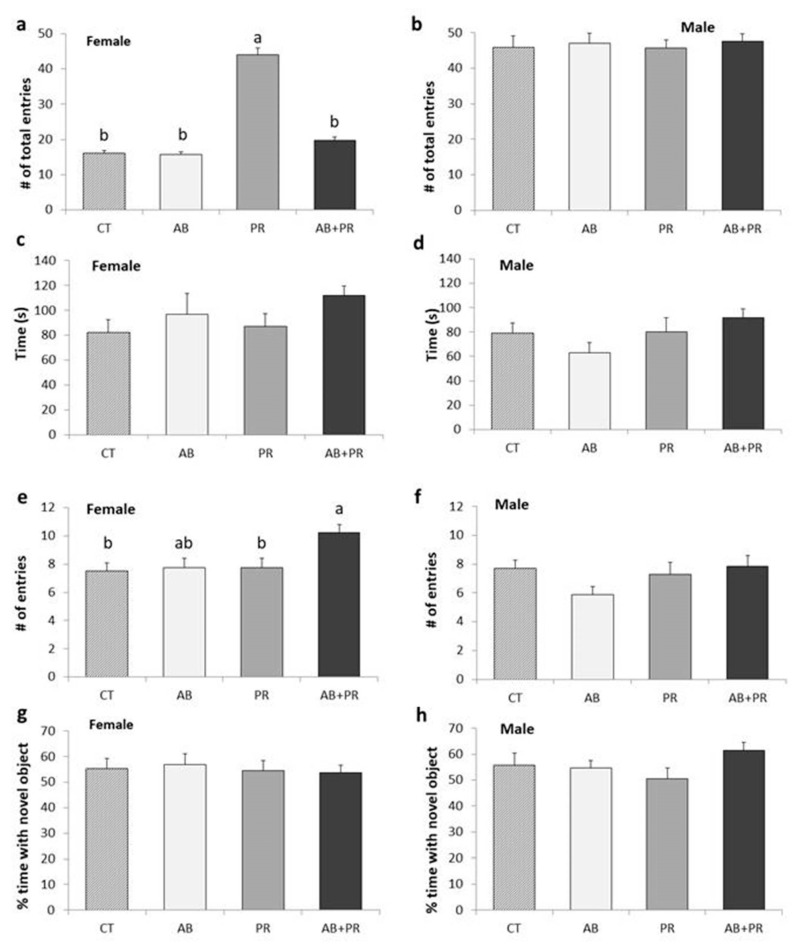
(**a**) Female total number of entries in open and closed arms; (**b**) male total number of entries; (**c**) female time in open arms; (**d**) male time in open arms; (**e**) female number of entries through open arms; (**f**) male number of entries through open arms; (**g**) female percent time with novel object; (**h**) male percent time with novel object. Values are mean ± SEM (*n* = 10–13/group). Post hoc analysis is depicted with the superscripts ^a,b^ where groups without a common superscript differ (*p* < 0.05) (i.e., a is different from b but ab is not different from a or b). CT: control; AB: antibiotic; PR: prebiotic; AB + PR: antibiotic plus prebiotic.

To examine the effects of maternal antibiotic/prebiotic intake on offspring behavior, we conducted the elevated plus maze (EPM) and novel context mismatch (NCM) tests. For the number of total entries into open and closed arms in the EPM, there was a significant main effect of sex (*p* = 0.03); therefore, sexes were considered separately. PR females showed more movement through the maze compared to all other groups, as seen by significantly greater total entries in and out of open and closed arms (*p* < 0.0001) (Figure 2a). Males showed no changes in total movement (Figure 2b). There were no significant differences in time spent in the open arm for females (Figure 2c) or males (Figure 2d). In females, the AB + PR group showed significantly higher number of entries into the open arms compared to PR (*p* = 0.024) and CT (*p* = 0.019), and there was a tendency towards more entries compared to AB although this was not significant (*p* = 0.059) (Figure 2e).

There were no significant differences in performances between groups in the NCM for females (Figure 2g) or males (Figure 2h).

### 3.3. Brain Gene Expression

We next examined the expression of genes involved in inflammatory responses in the brain. For Iba-1 expression, there was a significant main effect of LPS injection (*p* < 0.0001) and a significant main effect of sex (*p* < 0.0001); therefore, LPS-injected and saline-injected animals were analyzed separately, as were male and females. No maternal diet differences were detected for Iba-1 and there were no differences in Drd1 expression across all groups and brain regions (Supplementary Figure S1).

**Figure 3:**
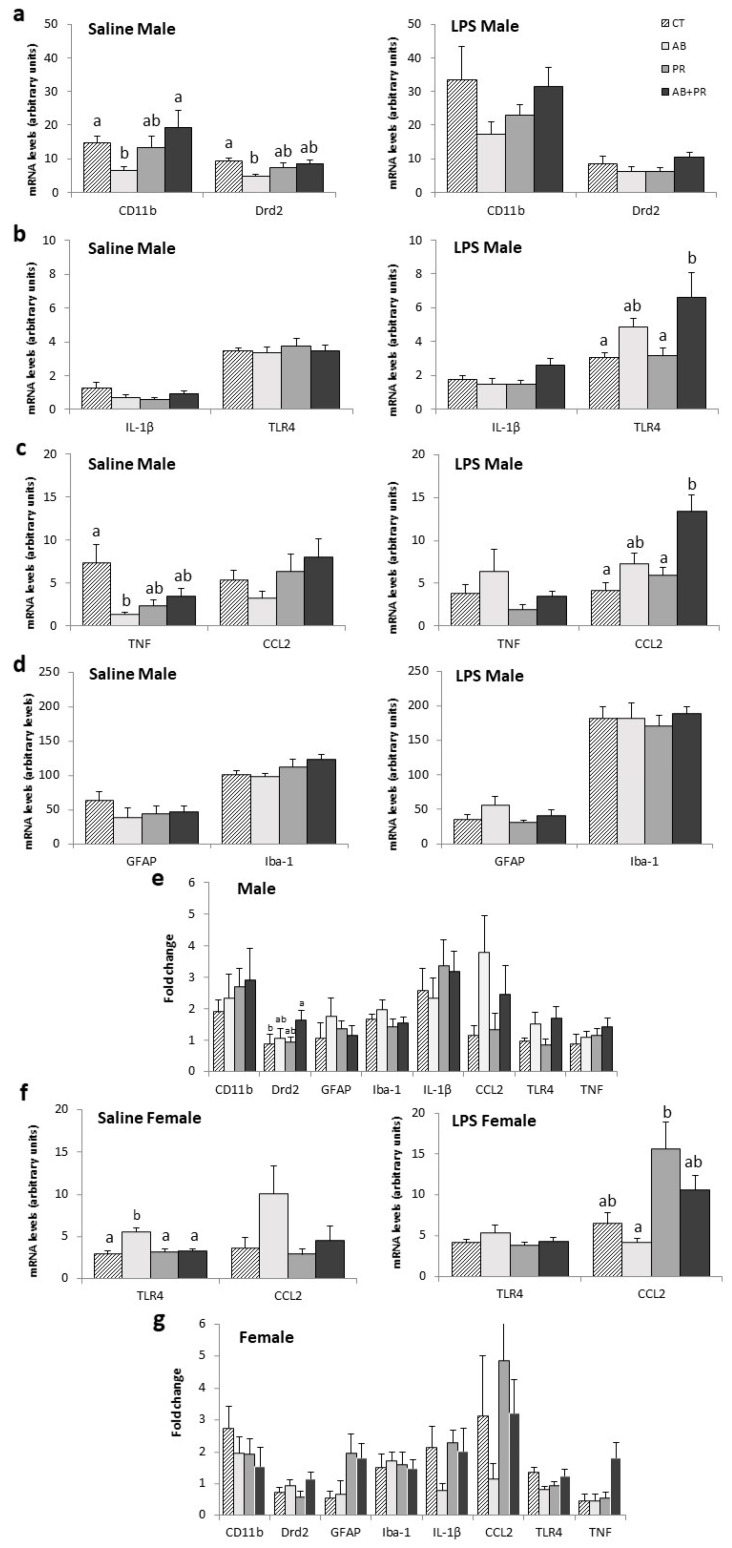
Gene expression in the hippocampus; (**a**) Saline- and lipopolysaccharide (LPS)-injected male mRNA levels of CD11b and Drd2; (**b**) saline- and LPS-injected male mRNA levels of IL-1β and TLR4; (**c**) saline- and LPS-injected male mRNA levels of TNF and CCL2; (**d**) Saline- and LPS-injected male mRNA levels of GFAP and Iba-1; (**e**) fold change in gene expression between LPS- and saline-injected males; (**f**) saline- and LPS-injected female mRNA levels of TLR4 and CCL2; (**g**) fold change in gene expression between LPS- and saline-injected females. Values are mean ± SEM (*n* = 10–13/group). Post hoc analysis is depicted with the superscripts ^a,b^ where groups without a common superscript differ (*p* < 0.05) (i.e., a is different from b but ab is not different from a or b). CT: control; AB: antibiotic; PR: prebiotic; AB + PR: antibiotic plus prebiotic.

Hippocampus: In saline-injected males, AB had lower levels of CD11b mRNA compared to AB + PR (*p* = 0.016) and CT (*p* = 0.034) (Figure 3a). AB also showed lower levels of Drd2 mRNA compared to CT (*p* = 0.009), and there was a tendency towards lower levels than AB + PR; however, this was not significant (*p* = 0.05) (Figure 3a). In LPS-injected males, AB + PR had higher TLR4 levels compared to CT (*p* = 0.031) and PR (*p* = 0.014) (Figure 3b). LPS-injected males also showed higher CCL2 mRNA levels in AB + PR compared to CT (*p* < 0.0001) and PR (*p* = 0.035) (Figure 3c), while saline-injected males showed lower TNF mRNA levels in AB compared to CT (*p* = 0.012) (Figure 3c). There were no differences in GFAP and Iba-1 mRNA levels in males (Figure 3d).

Comparing LPS injected to saline controls, AB + PR males had 1.64-fold increased expression of Drd2, which was significantly greater than the change seen in LPS versus saline injected CT (*p* = 0.026) (Figure 3e), but there were no other significant effects.

In saline-injected females, AB showed significantly higher levels of TLR4 mRNA compared to CT (*p* = 0.001), PR (*p* = 0.002) and AB + PR (*p* = 0.003) (Figure 3f). LPS-injected AB females had lower CCL2 mRNA levels compared to PR (*p* = 0.018) (Figure 3f). There were no significant differences between female groups for any of the other target genes (Supplementary Figure S2). The fold change in gene expression between LPS and saline-injected animals did not reach significance between groups in females (Figure 3g).

**Figure 4:**
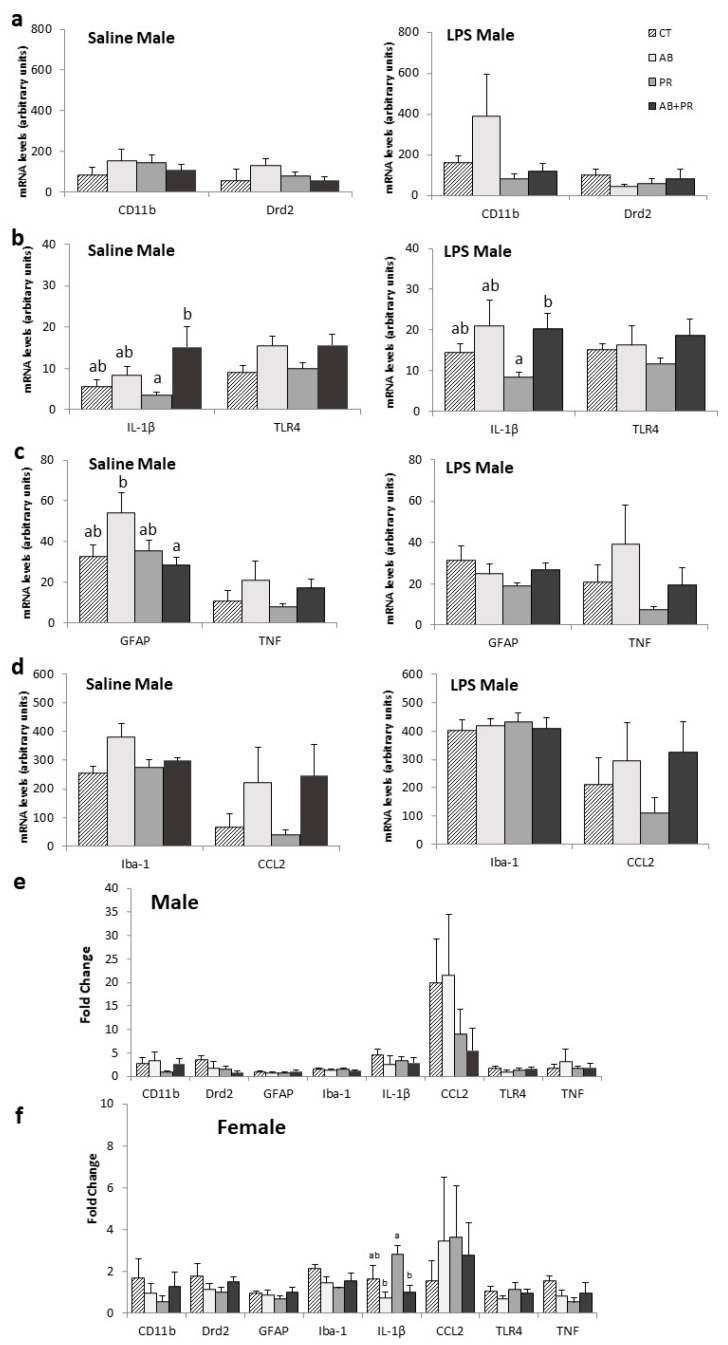
Gene expression in the hypothalamus; (**a**) saline- and LPS-injected male mRNA levels of CD11b and Drd2; (**b**) saline- and LPS-injected male mRNA levels of IL-1β and TLR4; (**c**) saline- and LPS-injected male mRNA levels of GFAP and TNF; (**d**) saline- and LPS-injected male mRNA levels of Iba-1 and CCL2; (**e**) fold change in gene expression between LPS- and saline-injected males; (**f**) fold change in gene expression between LPS- and saline-injected females. Values are mean ± SEM (*n* = 10–13/group). Post hoc analysis is depicted with the superscripts ^a,b^ where groups without a common superscript differ (*p* < 0.05) (i.e., a is different from b but ab is not different from a or b). CT: control; AB: antibiotic; PR: prebiotic; AB + PR: antibiotic plus prebiotic.

Hypothalamus: There were no differences between groups for CD11b and Drd2 expression in the hypothalamus (Figure 4a). In saline-injected males, PR showed lower IL-1β mRNA levels compared to AB + PR (*p* = 0.025) (Figure 4b). After LPS injection, males in the AB + PR group showed higher IL-1β mRNA levels than PR (*p* = 0.035) (Figure 4b). In saline-injected males, AB had higher GFAP mRNA levels compared to AB + PR (*p* = 0.032) (Figure 4c). There were no differences in Iba-1 and CCL2 in males (Figure 4d). The fold change in gene expression between LPS and saline-injected animals did not reach significance between groups in males (Figure 4e).

There were no significant differences in target genes between treatment groups in saline-injected females or LPS-injected females (Supplementary Figure S3). However, the fold change in the expression of IL-1β in LPS- compared to saline-injected animals was 2.82-fold in PR, which was significantly greater than AB (*p* = 0.012) and AB + PR (*p* = 0.029) groups (Figure 4f).

### 3.4. Microglia Analysis

Microglia were analyzed in the paraventricular nucleus of the hypothalamus (PVN) after LPS injection. Since there was a significant main effect of treatment (*p* < 0.0001) and LPS injection (*p* < 0.0001) but not sex, male and females were combined, and a one-way ANOVA was conducted to determine if there were differences between treatment groups in microglia count, average soma size, fluorescence intensity, process length, and number of intersections as determined by Sholl analysis. 

**Figure 5:**
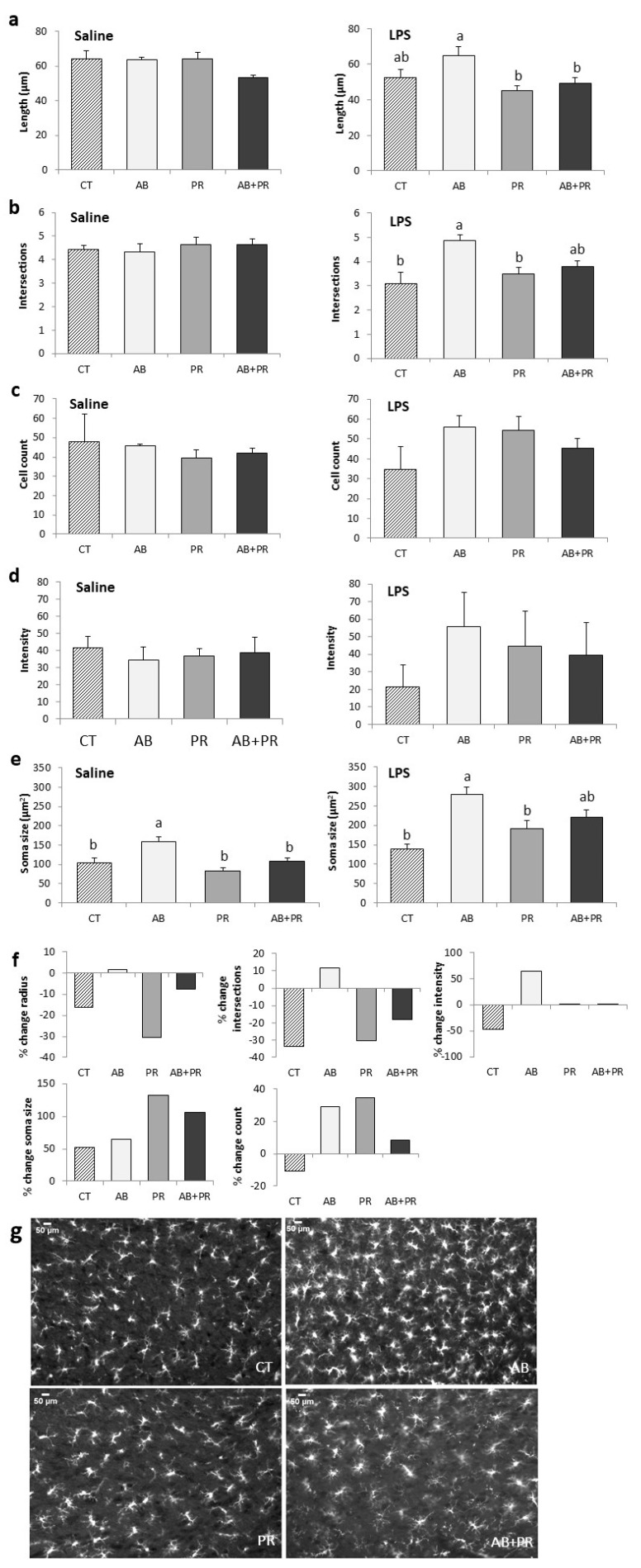
In the PVN (**a**) average length of longest process in saline- and LPS-injected rats; (**b**) average number of intersections as determined by Sholl analysis in saline- and LPS-injected rats; (**c**) cell count per 1.77 m^2^ in saline- and LPS-injected rats; (**d**) fluorescent intensity as assessed by mean grey value in saline- and LPS-injected rats; (**e**) average soma size (μm^2^) in saline- and LPS-injected rats; (**f**) average percent change in radius, intersections, cell count, intensity, and soma size in LPS- compared to saline-injected animals (means only); (**g**) representative images of LPS-injected immunofluorescent Iba-1 microglia under fluorescent microscopy in the PVN of the hypothalamus. Scale bars = 50 μm. Values are mean ± SEM (*n* = 3–6/group). Post hoc analysis is depicted with the superscripts ^a,b^ where groups without a common superscript differ (*p* < 0.05) (i.e., a is different from b but ab is not different from a or b). CT: control; AB: antibiotic; PR: prebiotic; AB + PR: antibiotic plus prebiotic.

In LPS-injected animals, the process lengths of AB offspring were significantly longer than PR (*p* = 0.007) and AB + PR (*p* = 0.039) (Figure 5a). LPS injection also revealed differences in microglial complexity in AB offspring compared to CT (*p* = 0.008) and PR (*p* = 0.012) (Figure 5b). AB showed a tendency towards greater complexity compared to AB + PR, but this was not significant (*p* = 0.079) (Figure 5b). There were no significant differences in cell count and fluorescence intensity between groups (Figure 5c,d). In saline-injected animals AB offspring had soma that were significantly larger than CT (*p* = 0.017), PR (*p* = 0.001), and AB + PR (*p* = 0.012) (Figure 5e). After LPS injection, AB soma sizes remained larger than CT (*p* = 0.002) and PR (*p* = 0.018) (Figure 5e). LPS lead to an overall percent decrease in radius length and number of intersections in all groups except AB (Figure 5f). CT was the only group that showed a decrease in cell count and intensity after LPS injection (Figure 5f). Representative images show the increase in soma size, complexity, and process length (Figure 5g).

### 3.5. Gut Gene Expression

We next examined the expression of intestinal barrier and inflammatory cytokine genes since inflammation of the gut or increased epithelial permeability are important drivers of peripherally driven CNS changes [47,48]. There was a significant main effect of sex on gut gene expression (*p* = 0.038); therefore, sexes were considered separately. Male and female offspring exposed to maternal antibiotics, prebiotics, both, or neither exhibited similar mRNA levels of tight junction proteins (occludin and ZO-1) as well as cytokines (TNF and IL-6) in the colon at 10 weeks of age (Supplementary Figure S4).

### 3.6. Cecal Bacterial Sequencing

**Figure 6:**
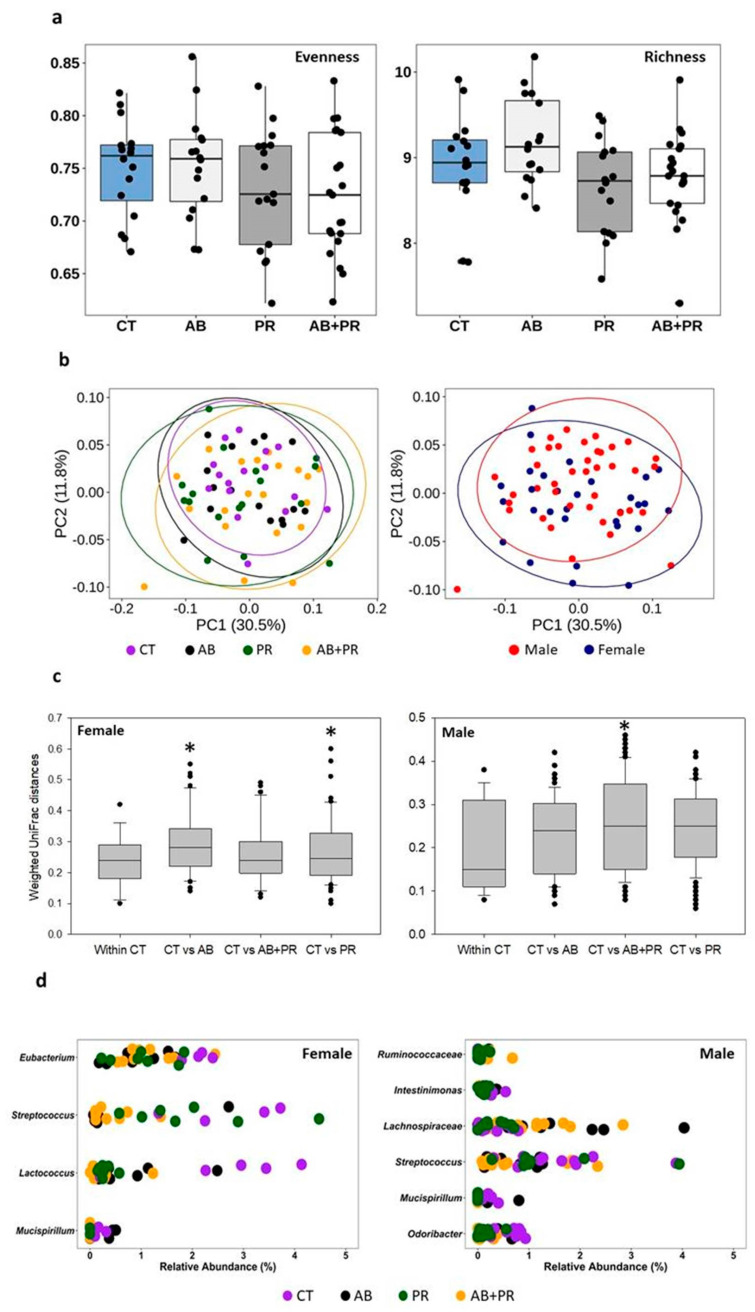
(**a**) Evenness and richness of cecal microbiota across different treatments; (**b**) PCoA plots of cecal microbiota calculated from UniFrac distance (weighted) matrix; (**c**) weighted UniFrac distance matrix-based analysis in females and males; (**d**) relative abundance across treatment groups in females and males. Values are mean ± SEM (*n* = 10–11/group). CT: control; AB: antibiotic; PR: prebiotic; AB + PR: antibiotic plus prebiotic.

To determine whether the inclusion of antibiotic/prebiotic in maternal diet affects offspring microbiota, cecal microbiota was analyzed by 16S rRNA gene sequencing. Maternal intake of low-dose penicillin and 10% OFS did not affect overall cecal microbiota of offspring at 10 weeks as measured by alpha diversity using evenness and richness (Figure 6a). Minor but significant differences were observed between female and male offspring in weighted UniFrac distance matrix-based analysis of community similarities (R = 0.097, *p* = 0.002) (Figure 6b). Dietary effects were identified as a significant contributor but showed variable patterns in female and male offspring (Figure 6c, R = 0.078, *p* = 0.004). In females, a significant difference was observed for AB (*p* = 0.014) and PR (*p* = 0.005) compared to CT (Figure 6c). Significant differences were also observed between CT and AB + PR in males (*p* = 0.033) (Figure 6c).

To determine which bacterial types were affected, the relative abundance of different bacterial types was compared separately in female and male offspring. Penicillin G is more active against gram-positive versus gram-negative bacteria [49] and despite the offspring not being exposed to the antibiotic, we saw a significant reduction in the relative abundance of Firmicutes in AB offspring compared to CT (Figure 6d). *Streptococcus*, a penicillin-sensitive genus, was significantly reduced in both female and male AB offspring. In female offspring, no difference was observed in Bacteroidetes and Actinobacteria. Other Firmicutes bacteria, including *Lactococcus* and *Eubacterium* were significantly decreased in both antibiotic groups (AB and/or AB + PR) compared to CT (Figure 6d). The abundance of *Mucispirillum*, a genus from the Deferribacteres phylum was also significantly decreased in AB + PR. Lower abundance of *Mucispirillum*, was detected in both prebiotic groups (PR and AB+PR) in male offspring. The only difference in Bacteriodetes was a reduction in *Odoribacter* in male AB + PR rats (Figure 6d). Variable differences in *Ruminococcaceae* abundance were observed in male offspring (Figure 6d).

## 4. Discussion

Maternal health is known to influence offspring gut microbiota colonization, which can have long-term effects on the offspring’s immune system, brain development, and overall health [2,6]. This study was designed to determine whether altering maternal microbiota during pregnancy and lactation with LDP and/or OFS would have long-term effects on offspring. We followed the design of a study that previously found perinatal exposure to low-dose penicillin led to long-term increases in weight gain and adiposity [25]. While they found that maternal antibiotic use alone was sufficient to induce metabolic effects, these were exacerbated by the addition of a high-fat diet [25]. We found that maternal exposure to antibiotics and prebiotics altered body composition, neuroimmune responses, and gene expression in the brain of their offspring despite no direct exposure by the offspring and no differences in gut-related gene expression in the offspring at 10 weeks. Antibiotic exposure of the dams impaired their offspring’s microglia reactivity to LPS and increased proinflammatory activity in the brain. Co-administering prebiotic OFS with the antibiotic in the dams was able to mitigate some of these detrimental effects, and most notably reversed the immature microglia phenotype.

Birth is a crucial period of vertical transmission of microbes from the mother to the infant, whereby the establishment of a healthy gut microbiota can have short- and long-term effects on the offspring’s health. In many developed countries, neonates are routinely exposed to prophylactic antibiotics for Group B *Streptococcus* infections, and this exposure has been shown to significantly affect the infant gut microbiota [50,51]. These changes include decreased diversity and richness, reduced abundance of Bacteroidetes, and greater relative abundance of Firmicutes at 3 months of age [51]. Although these microbial differences were less apparent by year 1 [51], the postnatal period remains critical for brain development, and these transient alterations in gut-brain communications may have long-lasting impacts. Contrasting with the tissue and organ development that occurs in utero, a vast amount of CNS development occurs postnatally, including synaptogenesis, cell differentiation, and the acquisition of function [52]. Events during pregnancy that alter offspring microbiota, including maternal antibiotic use, can affect microbiota and immunoregulation in offspring, which can then persist throughout life and lead to increased risk of neuropsychiatric disorders such as depression [5]. A study in mice found that antibiotic administration during gestation and lactation altered offspring microbiota at 4 and 14 days after birth including decreased levels of Bacteroidetes and increased Proteobacteria, which mimicked changes in the dams’ microbiota [3]. In preclinical models, pups exposed to the antibiotic vancomycin during gestation and lactation or lactation alone had reduced α-diversity at 14 days of age, and had altered immunity as seen by higher splenic cell counts and altered B cell counts [3]. Our lab has previously found that using the current protocol, LDP and OFS supplementation in dams altered offspring microbiota at weaning, and despite the microbial differences not persisting into adulthood, the antibiotic-associated obese phenotype remained [53]. 

In our study, maternal OFS intake was associated with significantly lower body weight in their offspring, despite offspring across all groups consuming similar caloric intake. This is comparable to previous work showing that maternal OFS consumption reduced body weight and fat mass in offspring at weaning [54] and attenuated hepatic steatosis in offspring fed a high fat/sucrose diet for 23 weeks [55]. Previous studies have demonstrated that prebiotics improve calcium absorption and increase bone mineral content and density [56,57,58]. While we did not see this enhanced bone mineral content in our PR rats, this is most likely because the offspring did not directly consume the prebiotic, only the dams did. The AB offspring had the highest bone mineral content, which could be due to this group having the highest body weight which would increase loading of the bones [59]; however, this was not significant likely indicating the effect was not substantial.

In the steatosis study mentioned above [55], gut microbiota differences in prebiotic versus control offspring were most prominent at 3 weeks of age, diminished by 11 weeks of age and absent by 24 weeks of age [55]. Cox et al. also found that after cessation of direct LDP, differences in microbial populations were transient, whereas the effects on various phenotypes such as body weight and obesity risk remained throughout life [25]. We found that maternal antibiotic use led to a significant decrease in the abundance of Firmicutes compared to CT offspring, including *Streptococcus*, *Lactococcus*, and *Eubacterium*. While an increase in *Bifidobacterium* would be expected from direct consumption of prebiotics, it is important to note that our animals were not directly exposed to prebiotics and the influence of prebiotic-altered microbiota on the offspring would have been via vertical transmission from the dams. It should also be considered that the cessation of breastfeeding/suckling drives major compositional changes in the gut microbiota, including a reduction in *Bifidobacterium* [60]. Therefore, while there may have been an increase in bifidobacteria before weaning, this difference was not apparent by 10 weeks of age when we assessed gut microbiota composition. Additionally, our offspring were given an HFS diet, which has also been shown to decrease bifidobacteria [61], and may have overridden any potential early bifidogenic profile. A recent clinical study found that the effects of antibiotic exposure during the first year of life on microbiota were not sustained, showing no differences in diversity measures across the first 4 years of life [62]. Similarly, a study that employed a randomized exposure to either placebo or azithromycin in children around the age of two also showed that antibiotic administration reduced richness and Shannon diversity short-term (14 days), but these differences were no longer seen 13–39 months after their last treatment [63]. A study in Finnish school children found that macrolide use but not necessarily penicillin-type antibiotic use was associated with long-term changes in microbiota composition and function [64]. These studies suggest that although major changes in gut microbiota may not persist into childhood or adolescence, the perturbation of microbiota during critical early life periods may have lasting effects, as we found in offspring gene expression in the brain and microglial morphology.

While the physical phenotype of offspring exposed to maternal antibiotic treatment has been described in several previous studies, examination of the behavioral outcomes associated with early life antibiotic and/or prebiotic exposure is far less common. In the current work, maternal prebiotic supplementation alongside antibiotics resulted in increased open arm activity, but otherwise led to limited behavioral changes. A previous study in mice showed that antibiotics induced anxiety-like behavior, which was then reversed following treatment with lactobacilli [65], whereas we did not see such strong effects of antibiotics on behavior with our subtherapeutic antibiotic dose. A systematic review of clinical trials found that the effect of prebiotics did not differ compared to placebo in their treatment for anxiety and depression [66]. However, it should be noted that the interventions included in the systematic review had an age range of 20–70 years [66]. It is well-established that early life is a critical period of development (prenatal to 3 years of age), and dysbiosis in early life could lead to long-term alterations in brain function and risk of psychiatric disorders [67]. Leclercq et al. found that feeding oral low-dose penicillin in BALB/c mice pre- and postnatally reduced anxiety-like behaviors in males only [68]. Another study examining juvenile male rats found that a diet including a blend of prebiotics reduced learned helplessness behaviors and reduced anxiety and stress response [69]. While there are limited studies on the effects of prebiotics and anxiety disorders, our study is the first to suggest that early-life indirect exposure to prebiotics in combination with antibiotics resulted in limited anxiety-like behavior later in life compared to exposure to antibiotics alone.

The hippocampus is related to temporal and spatial memory, and has a role in food intake [70]. We found a reduction of Drd2 mRNA levels in AB saline-injected males compared to CT; however, this was not correlated with increased food intake, and only with a trend towards increased body weight. Inhibition of hippocampal Drd2^+^ neurons was shown to lead to increased food intake and Drd2 expression was linked to food-related memory associations in mice [70]. We also saw a sex difference in both expression of TLR4 in the hippocampus and in behavior as seen in the EPM. In males, we saw a significant increase in AB + PR hippocampal TLR4 in response to an LPS injection compared to CT and PR alone. This was not seen in saline-injected males, indicating that the peripheral inflammation may have unmasked a primed immune response in the AB + PR males. A previous study found that suppressing TLR4 expression is protective against age-related anxiety-like behavior in a sex-dependent manner [71]. We also found that AB + PR females showed more movement into the open arms of the EPM, whereas there were no differences in males. TLR4 inhibition during development has been found to improve hippocampus-dependent spatial, contextual, and motor learning, whereas TLR4 inhibition during adulthood alters anxiety-like behavior as assessed by the open field and elevated plus maze [72]. While it is unclear why these sex-dependent differences were seen, they could be related to previously described sex differences in gut microbiota composition in mice and perhaps more importantly by sex-by-diet interactions where depending on the sex of the mice, microbial response to chow or high fat diet differed [73].

We found that after LPS injection AB + PR males also had higher levels of CCL2 mRNA compared to CT and PR. Peripheral inflammation can lead to a central immune response, which is often initiated by glial cells such as astrocytes and microglia [74]. CCL2 mRNA has been found to be predominantly expressed by astrocytes in the hippocampus after ischemia-reperfusion in spontaneously hypertensive rats [75]. The increase in CCL2 in AB + PR males after peripheral inflammation could indicate that this group had an increased immune response, indicating that prebiotic supplementation may not be uniformly beneficial. This immune response could include increased astrocyte activity, microglia recruitment, and possibly increased numbers of infiltrating monocytes [75]. GFAP is another signature marker of astrocyte activity in the brain. We found that GFAP mRNA levels were increased in the hypothalamus of AB males who were saline-injected compared to CT. Interestingly, GFAP was also found to increase in the hypothalamus in mice as dietary fat content increased, alongside reduced insulin sensitivity and alterations in hippocampal-dependent memory [76]. The effect of our HFS diet may have led to an expected increase in hypothalamic GFAP expression, which was protected by maternal supplementation with prebiotics, similar to how prebiotic fiber supplementation has been linked to reduced adiposity and improved insulin sensitivity [27]. An increased number of microglia in the arcuate nucleus (ARC) of the hypothalamus has been shown to increase body weight gain and increase fat mass, which can all be reversed by blocking the proliferation of microglia in these regions [77]. Therefore, it could be that by reducing weight gain and improving metabolic parameters, prebiotic supplementation also reduces associated inflammation in brain regions key to stress response, such as the PVN of the hypothalamus. 

Studies in germ free (GF) mice show that a lack of microbiota increases Iba-1 in the hypothalamus as well as increases the size of microglia [78]. These changes were only seen in the hypothalamus, and not in other brain regions such as the hippocampus [78]. Peripheral inflammation is associated with a central inflammatory response, mediated by the activation of microglia [40]. Morphologically, microglia in a ‘resting’ state show a small soma connected to branched cellular processes [37]. In an activated state, microglia reduce their complexity, retract their processes, and develop an ameboid-like phenotype. Here we show that early life antibiotic exposure during a critical developmental window altered microglia activation in response to a peripheral immune challenge. Indirect exposure to antibiotics in early life led to altered responses to LPS challenge, which was attenuated with the co-administration of prebiotics. We found AB offspring showed an increased density of microglia and increased soma size compared to CT, which was reduced in AB + PR offspring. We also found the process length and complexity was significantly increased in AB compared to PR offspring after an immune challenge. Our results are in line with the findings by Erny et al. [8], who found that germ-free and antibiotic-treated mice showed a diminished microglia response to LPS infection. They found that SCFA administration was able to reverse the immature microglia phenotype [8]. Another previous study found that a high-fat diet led to altered microglia, as seen by increased soma size, reduced process length, and increased Iba-1 positive cells compared to a normal diet [79]. The prebiotic xylooligosaccharide, the probiotic *Lactobacillus paracasei* HIIO1, or a combined synbiotic reversed all microglial parameters in the hippocampus of male Wistar rats [79]. Our microglia analysis focused on the PVN, which is part of the hypothalamic-pituitary-adrenal (HPA) axis and is considered the major neuroendocrine system that regulates the body’s response to stress [80]. Since microglia are crucial in early life for synaptic pruning, maternal antibiotic use could alter the rates of synaptogenesis during this critical window of development, which then persists long after the offspring have been weaned [81,82]. Here, we found that maternal antibiotic use leads to altered neuroimmune responses in their offspring at 10 weeks of age, as seen by longer processes and greater dendrite complexity in response to LPS. Using prebiotics as a dietary adjunct to antibiotic use in dams was enough to prevent this phenotype, and these offspring showed a neuroimmune response comparable to the control group. 

To our knowledge, this is the first paper exploring maternal antibiotic use and its effect on offspring behavior, gut microbiota, and neuroimmune responses, and the potential mitigation of adverse effects with maternal prebiotic consumption. A limitation of this study is that we did not analyze SCFA, which are microbial metabolites of prebiotics that are known to directly influence microglia [8]. Furthermore, we are not able to determine which time point is most critical for antibiotic exposure in altering the brain, since our antibiotics were given during both gestation and lactation. Studies moving forward should isolate antibiotic doses to gestation or lactation only to elucidate the effects of microbial dysbiosis during specific timepoints. Due to the small sample size in our microglia analysis subgroup, we did not stratify microglia analysis by sex. Given that the gastrointestinal tract is a major immune organ that is highly influenced by sex hormones, which can alter gut-brain interactions [83], larger-scale studies should aim to analyze the effects of early life microbiota exposures and sex-specific differences in microglia. Future studies should also employ fecal microbiota transplantation to determine the causative role altered gut microbiota in maternal antibiotic and prebiotic supplementation on neuroimmune responses.

## 5. Conclusions

Healthy early life gut microbiota is critical for the development of healthy nervous and immune systems, and can be perturbed by perinatal antibiotic use. Indirect exposure to antibiotics/prebiotics via maternal intake was demonstrated to alter weight gain, gene expression in the brain, and microglia reactivity to peripheral inflammation in offspring. Maternal co-administration of prebiotic with antibiotic was shown to reduce body weight and increase CCL2 in the hippocampus, while reversing the antibiotic-associated dampened microglia response in the paraventricular nucleus of the hypothalamus. In this preclinical model, prebiotics show promise as a potential dietary adjunct to maternal antibiotic use during pregnancy and lactation, which, while increasingly used with antibiotic stewardship in mind, will continue to be clinically indicated in many cases.

## Figures and Tables

**Table 1:** Body composition and cecal and brain weight in female and male offspring at 10 weeks of age ^1^.

	CT	AB	PR	AB + PR	*p*-Value
Females					
Total Weight (g)	278.3 ± 7.2 ^a,b^	307.3 ± 8.0 ^a^	268.4 ± 9.3 ^b^	276.0 ± 4.0^b^	0.006
Lean + BMC (g)	216.9 ± 5.5	225.9 ± 6.7	210.9 ± 5.9	217.0 ± 5.5	0.361
Fat Mass (g)	65.2 ± 6.7 ^a,b^	81.4 ± 6.6 ^a^	57.5 ± 4.6^b^	56.7 ± 4.0^b^	0.009
% Body Fat	22.7 ± 1.6 ^a,b^	26.3 ± 1.8 ^a^	21.1 ± 1.2 ^a,b^	20.5 ± 1.4^b^	0.038
Cecum weight/body weight (g)	0.00149 ± 0.0001	0.00145 ± 0.0001	0.00173 ± 0.0001	0.00166 ± 0.0000	0.068
Brain weight/body weight (g)	0.00709 ± 0.0002	0.00647 ± 0.0001	0.00718 ± 0.0003	0.00709 ± 0.0001	0.056
Bone Mineral Content (g)	7.49 ± 0.25	7.80 ± 0.24	7.39 ± 0.22	7.81 ± 0.23	0.454
Bone Mineral Density (g/cm^2^)	0.134 ± 0.002	0.137 ± 0.001	0.136 ± 0.002	0.140 ± 0.002	0.099
Males					
Total Weight (g)	460.1 ± 9.6 ^a,b^	487.4 ± 7.0 ^b^	434.9 ± 14.7 ^a^	445.2 ± 8.2 ^a^	0.010
Lean + BMC (g)	361.7 ± 5.4 ^a,b^	380.2 ± 4.4 ^b^	343.0 ± 11.2 ^a^	356.0 ± 4.5 ^a,b^	0.011
Fat Mass (g)	98.4 ± 7.0	107.1 ± 4.3	92.0 ± 7.5	89.2 ± 6.0	0.242
% Body Fat	21.2 ± 1.2	21.9 ± 0.7	20.9 ± 1.4	19.9 ± 1.1	0.679
Cecum weight/body weight (g)	0.00119 ± 0.0001 ^a^	0.00121 ± 0.0000 ^a,b^	0.00139 ± 0.0000 ^b^	0.00136 ± 0.0000 ^a,b^	0.013
Brain weight/body weight (g)	0.00467 ± 0.0001	0.00447 ± 0.0001	0.00494 ± 0.0002	0.00462 ± 0.0002	0.166
Bone Mineral Content (g)	10.3 ± 0.3	10.7 ± 0.2	9.9 ± 0.3	10.6 ± 0.3	0.220
Bone Mineral Density (g/cm^2^)	0.140 ± 0.002	0.142 ± 0.001	0.140 ± 0.001	0.142 ± 0.002	0.644

^1^ Values are means ± SEM, *n* = 10–13. The *p*-value refers to the one-way ANOVA outcome. Post hoc analysis is depicted with the superscripts ^a,b^ where groups without a common superscript differ (*p* < 0.05) (i.e., a is different from b but ab is not different from a or b). CT: control; AB: antibiotic; PR: prebiotic; AB + PR: antibiotic plus prebiotic; BMC: bone mineral content.

## Supplementary Materials

The following are available online at https://www.mdpi.com/2076-2607/8/7/1085/s1, Figure S1. Gene expression of Drd1 in the hippocampus and hypothalamus of females and males injected with saline or LPS. Values are mean ± SEM (n = 10–13/group). No significance. CT: control; AB: antibiotic; PR: prebiotic; AB + PR: antibiotic plus prebiotic, Figure S2. Gene expression in the hippocampus of females injected with saline or LPS. Values are mean ± SEM (n = 10–13/group). No significance. CT: control; AB: antibiotic; PR: prebiotic; AB + PR: antibiotic plus prebiotic, Figure S3. Gene expression in the hypothalamus of females injected with saline or LPS. Values are mean ± SEM (n = 10–13/group). No significance. CT: control; AB: antibiotic; PR: prebiotic; AB + PR: antibiotic plus prebiotic, Figure S4. (a) Female offspring mRNA levels of occludin and tight junction protein ZO-1; (b) male offspring mRNA levels of occludin and tight junction protein ZO-1; (c) female offspring mRNA levels of TNF and IL-6; (d) male offspring mRNA levels of TNF and IL-6. Values are mean ± SEM (*n* = 10–11/group). No significant differences. CT: control; AB: antibiotic; PR: prebiotic; AB + PR: antibiotic plus prebiotic.
