# Supplementary material for: Impaired Hypothalamic Microglial Activation in Offspring of Antibiotic-Treated Pregnant/Lactating Rats Is Attenuated by Prebiotic Oligofructose Co-Administration
Source: Microorganisms. 2020 Jul 21;8(7):1085. doi: 10.3390/microorganisms8071085 (PMC7409116; doi:10.3390/microorganisms8071085)
Supplement: Supplementary file 1 [file microorganisms-08-01085-s001.pdf]

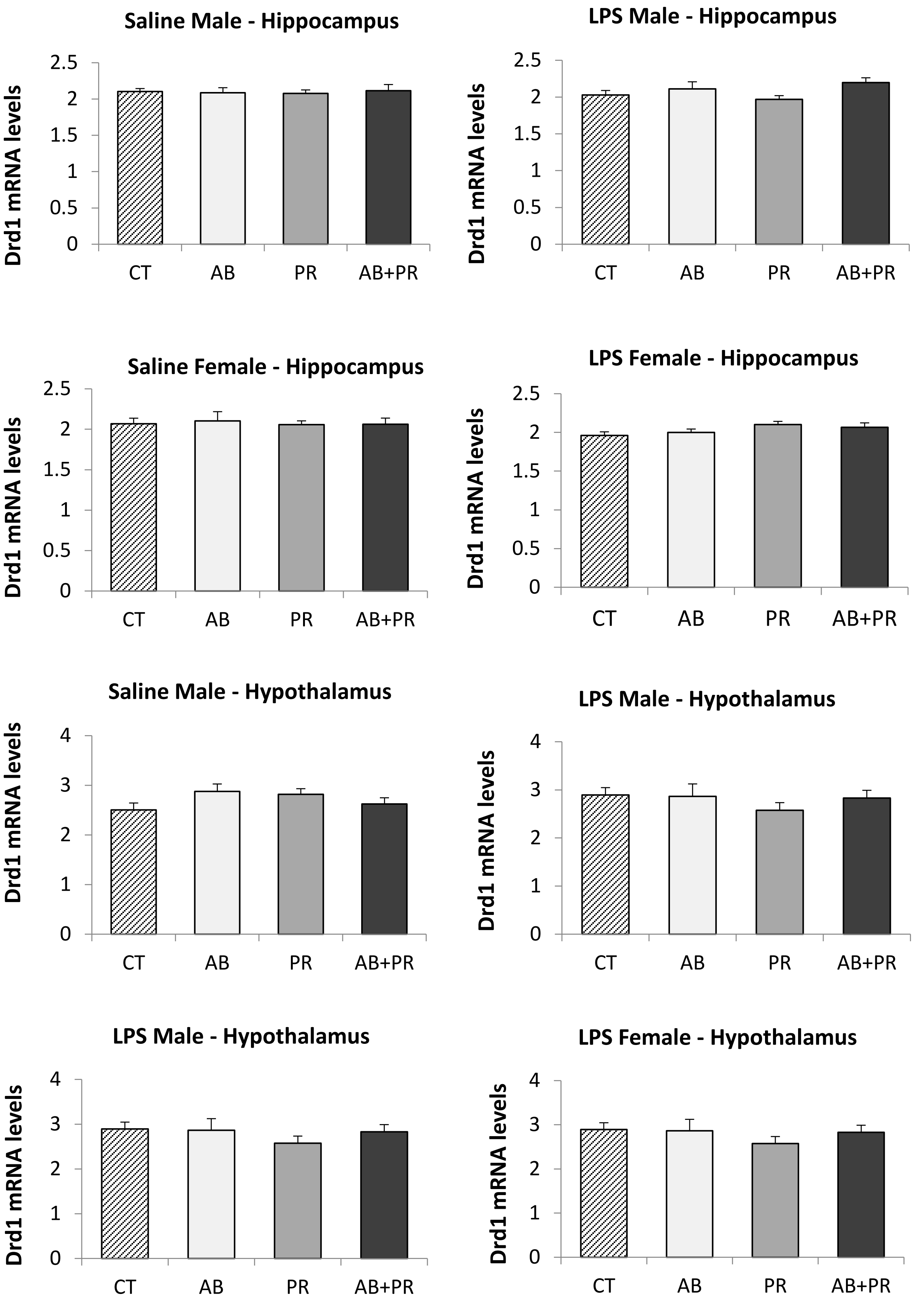

Figure S1. Gene expression of Drd1 in the hippocampus and hypothalamus of females and males injected with saline or LPS. Values are mean ± SEM (n=10-13/group). No significance. CT: control; AB: antibiotic; PR: prebiotic; AB + PR: antibiotic plus prebiotic.

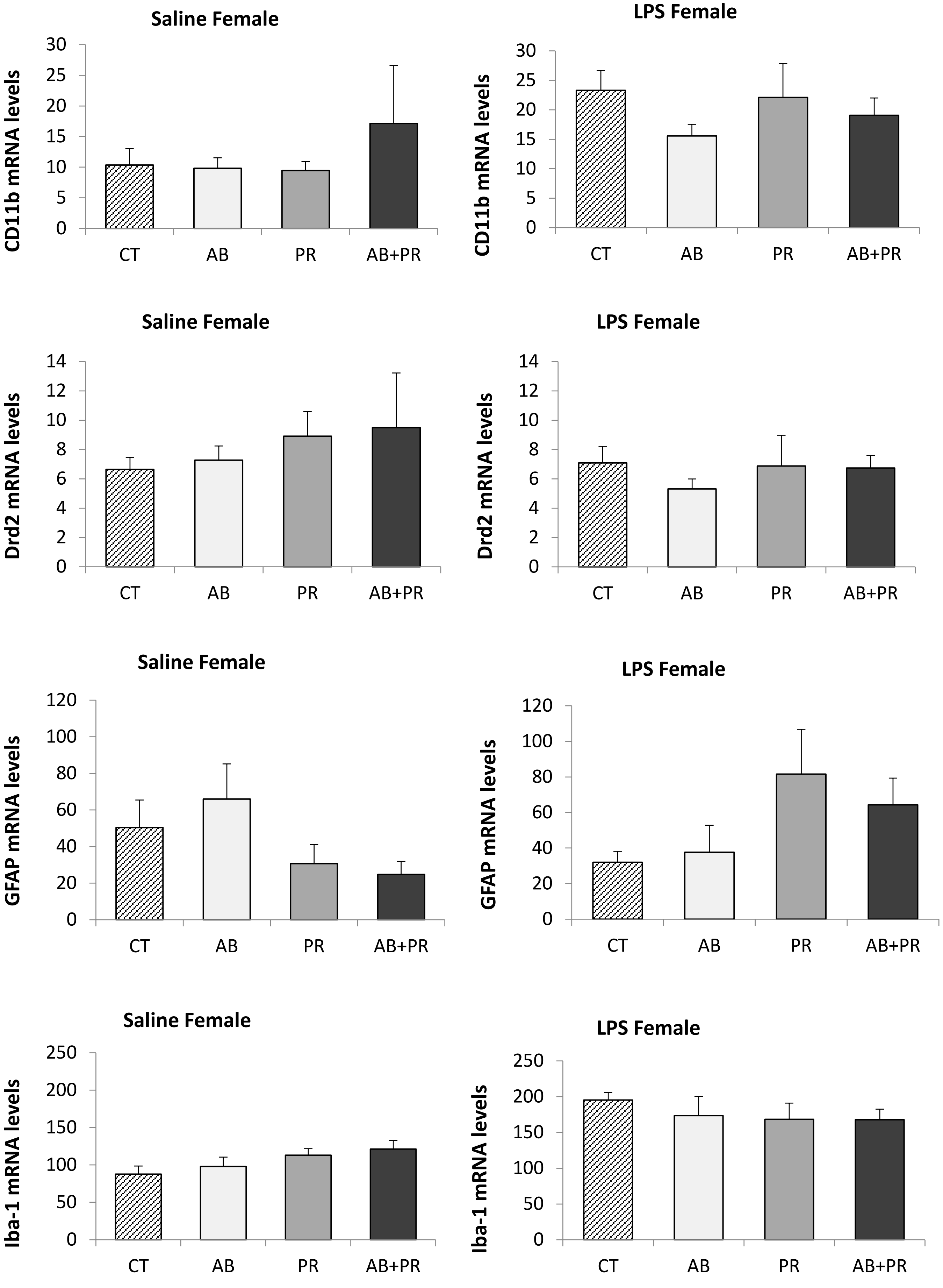

Saline Female
CD11b mRNA levels
0
5
10
15
20
25
30
CT
AB
PR
AB+PR
LPS Female
CD11b mRNA levels
0
5
10
15
20
25
30
CT
AB
PR
AB+PR
Saline Female
Drd2 mRNA levels
0
2
4
6
8
10
12
14
CT
AB
PR
AB+PR
LPS Female
Drd2 mRNA levels
0
2
4
6
8
10
12
14
CT
AB
PR
AB+PR
Saline Female
GFAP mRNA levels
0
20
40
60
80
100
120
CT
AB
PR
AB+PR
LPS Female
GFAP mRNA levels
0
20
40
60
80
100
120
CT
AB
PR
AB+PR
Saline Female
Iba-1 mRNA levels
0
50
100
150
200
250
CT
AB
PR
AB+PR
LPS Female
Iba-1 mRNA levels
0
50
100
150
200
250
CT
AB
PR
AB+PR

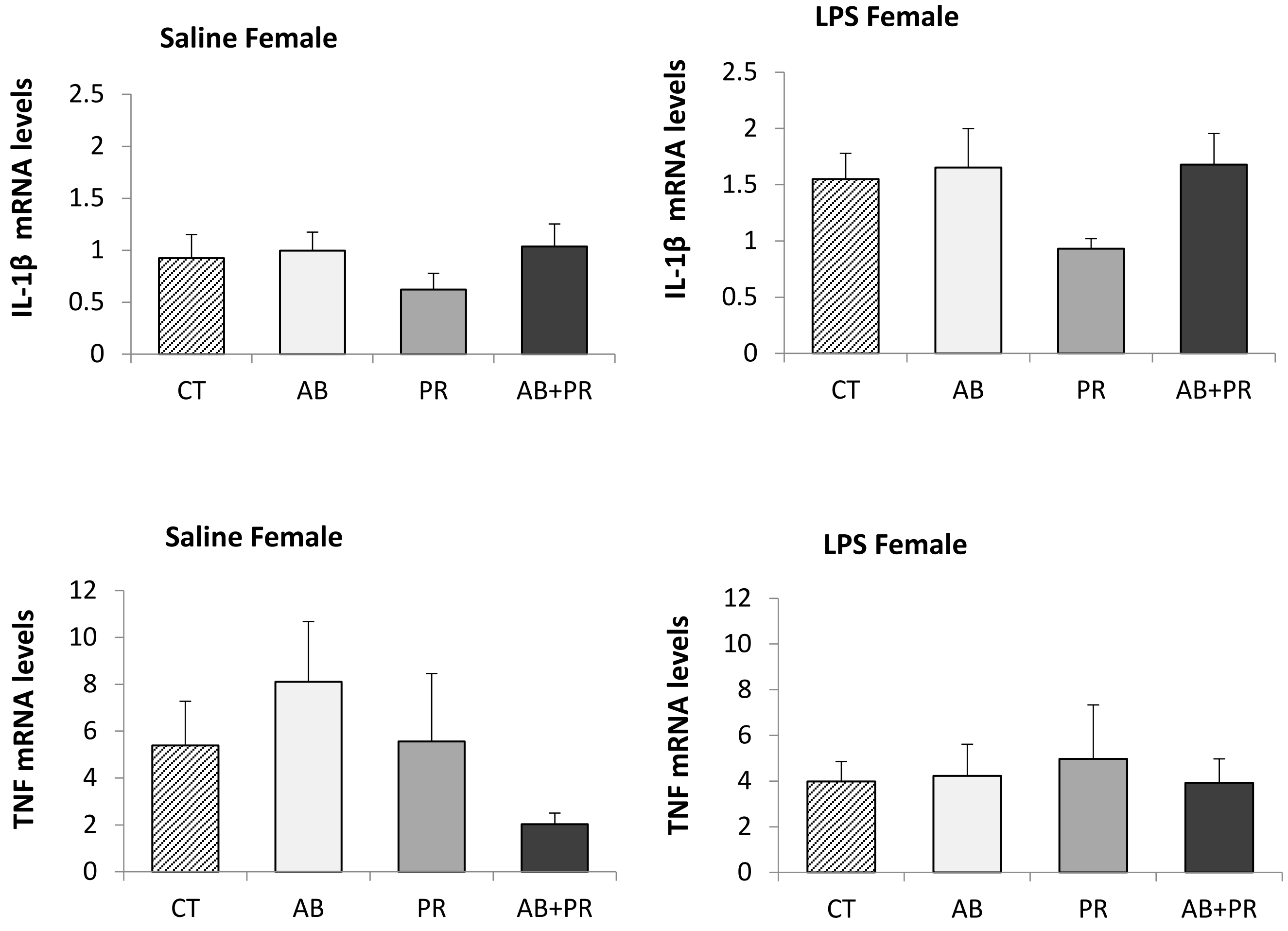

Figure S2. Gene expression in the hippocampus of females injected with saline or LPS. Values are mean ± SEM (n=10-13/group). No significance. CT: control; AB: antibiotic; PR: prebiotic; AB + PR: antibiotic plus prebiotic.

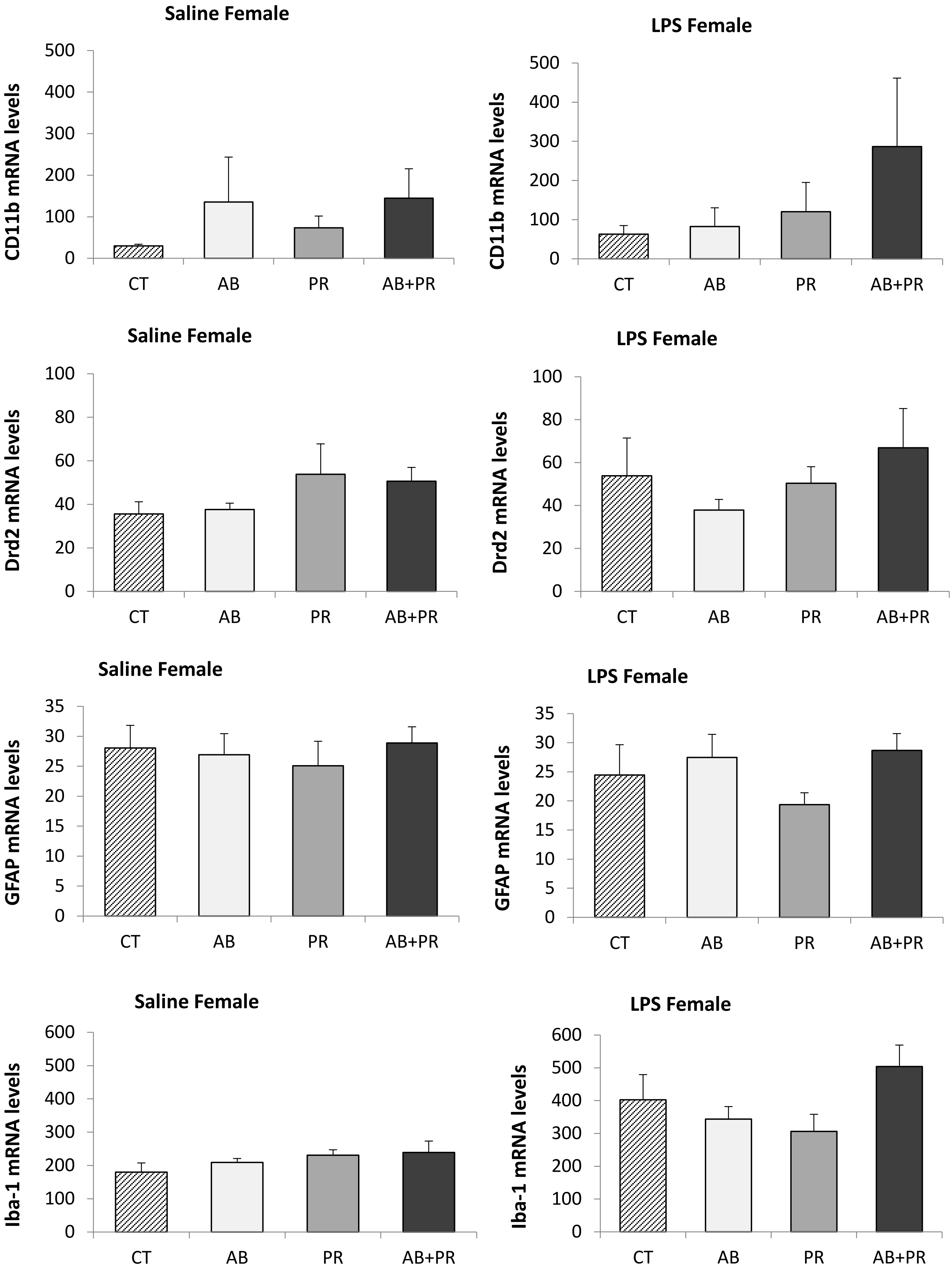

Saline Female
CD11b mRNA levels
500
400
300
200
100
0
CT
AB
PR
AB+PR
LPS Female
CD11b mRNA levels
500
400
300
200
100
0
CT
AB
PR
AB+PR
Saline Female
Drd2 mRNA levels
100
80
60
40
20
0
CT
AB
PR
AB+PR
LPS Female
Drd2 mRNA levels
100
80
60
40
20
0
CT
AB
PR
AB+PR
Saline Female
GFAP mRNA levels
35
30
25
20
15
10
5
0
CT
AB
PR
AB+PR
LPS Female
GFAP mRNA levels
35
30
25
20
15
10
5
0
CT
AB
PR
AB+PR
Saline Female
Iba-1 mRNA levels
600
500
400
300
200
100
0
CT
AB
PR
AB+PR
LPS Female
Iba-1 mRNA levels
600
500
400
300
200
100
0
CT
AB
PR
AB+PR

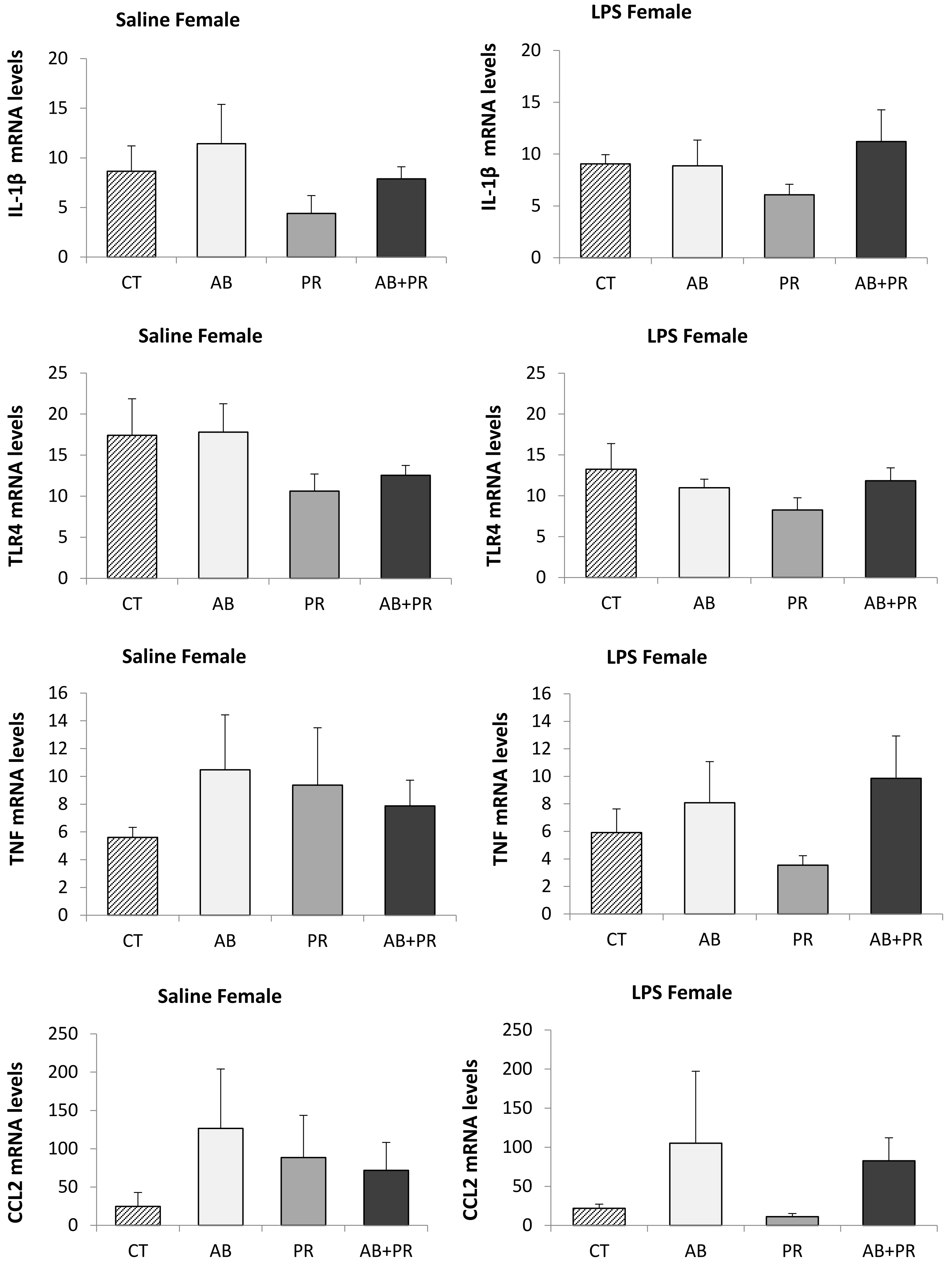
Saline Female
IL-1β mRNA levels
20
15
10
5
0
CT
AB
PR
AB+PR
LPS Female
IL-1β mRNA levels
20
15
10
5
0
CT
AB
PR
AB+PR
Saline Female
TLR4 mRNA levels
25
20
15
10
5
0
CT
AB
PR
AB+PR
LPS Female
TLR4 mRNA levels
25
20
15
10
5
0
CT
AB
PR
AB+PR
Saline Female
TNF mRNA levels
16
14
12
10
8
6
4
2
0
CT
AB
PR
AB+PR
LPS Female
TNF mRNA levels
16
14
12
10
8
6
4
2
0
CT
AB
PR
AB+PR
Saline Female
CCL2 mRNA levels
250
200
150
100
50
0
CT
AB
PR
AB+PR
LPS Female
CCL2 mRNA levels
250
200
150
100
50
0
CT
AB
PR
AB+PR

Figure S3. Gene expression in the hypothalamus of females injected with saline or LPS. Values are mean ± SEM (n=10-13/group). No significance. CT: control; AB: antibiotic; PR: prebiotic; AB + PR: antibiotic plus prebiotic.

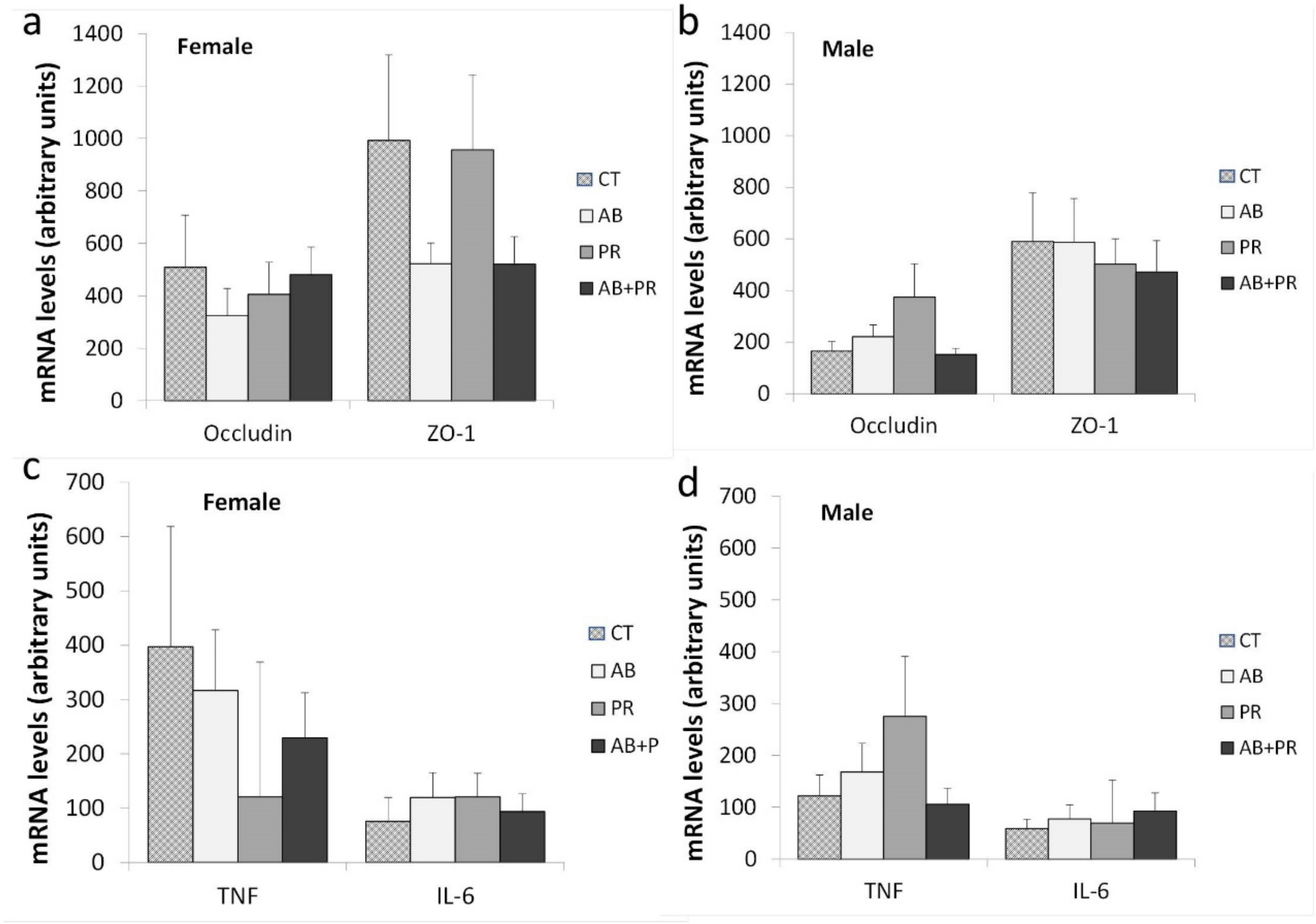

Figure S4. (**a**) Female offspring mRNA levels of occludin and tight junction protein ZO-1; (**b**) Male offspring mRNA levels of occludin and tight junction protein ZO-1; (**c**) Female offspring mRNA levels of TNF and IL-6; (**d**) Male offspring mRNA levels of TNF and IL-6. Values are mean ± SEM (n=10-11/group). No significant differences. CT: control; AB: antibiotic; PR: prebiotic; AB + PR: antibiotic plus prebiotic.
